# SuperPred: update on drug classification and target prediction

**DOI:** 10.1093/nar/gku477

**Published:** 2014-05-30

**Authors:** Janette Nickel, Bjoern-Oliver Gohlke, Jevgeni Erehman, Priyanka Banerjee, Wen Wei Rong, Andrean Goede, Mathias Dunkel, Robert Preissner

**Affiliations:** 1 Charité—University Medicine Berlin, Structural Bioinformatics Group, Institute of Physiology & Experimental Clinical Research Center, Berlin 13125, Germany; 2 Charité—University Medicine Berlin, Division of General Pediatrics, Department of Pediatric Oncology and Hematology, Berlin 13353, Germany; 3 German Cancer Consortium (DKTK), German Cancer Research Center (DKFZ), Heidelberg 69120, Germany; 4 Graduate School of Computational System Biology, Berlin 10115, Germany

## Abstract

The SuperPred web server connects chemical similarity of drug-like compounds with molecular targets and the therapeutic approach based on the similar property principle. Since the first release of this server, the number of known compound–target interactions has increased from 7000 to 665 000, which allows not only a better prediction quality but also the estimation of a confidence. Apart from the addition of quantitative binding data and the statistical consideration of the similarity distribution in all drug classes, new approaches were implemented to improve the target prediction. The 3D similarity as well as the occurrence of fragments and the concordance of physico-chemical properties is also taken into account. In addition, the effect of different fingerprints on the prediction was examined. The retrospective prediction of a drug class (ATC code of the WHO) allows the evaluation of methods and descriptors for a well-characterized set of approved drugs. The prediction is improved by 7.5% to a total accuracy of 75.1%. For query compounds with sufficient structural similarity, the web server allows prognoses about the medical indication area of novel compounds and to find new leads for known targets. SuperPred is publicly available without registration at: http://prediction.charite.de.

## INTRODUCTION

The Anatomical Therapeutic Chemical (ATC) classification system of the World Health Organization (WHO) is currently the most prevalent system to characterize drugs. This system is divided into several hierarchical categories differentiating between anatomical, therapeutic, pharmacological and chemical properties (1). Drug utilization can be investigated using the ATC classification system. Therefore, comparing the drugs’ structural and physico-chemical features by means of ATC codes offers a possibility to gain knowledge for drug repositioning and predicting new medical indications as well as classifying yet unclassified compounds. The established ‘similarity property principle’ (2) is based on the assumption that structurally similar molecules exhibit similar biological activity (3). Various 2D methods have been developed to search for similarity between compounds (4). Among others, topological descriptors like 2D fingerprints (5) or BCUT descriptors (6) are often applied in similarity searching. Although 2D fingerprints are widely used for various applications like virtual screening, similarity searching and clustering, several problems can occur. For instance, the molecular size of a compound can affect the similarity calculations as well as a folding of fixed-length bit strings which can result in the negligence of functional and structural features. To overcome these interferences, the SuperPred update (SuperPred II) does not only consider 2D similarity methods but also fragment and 3D similarity searching. Recently, some attempts have been undertaken to address the ATC prediction problem. Gurulingappa *et al.* used a combination of information extraction and machine learning techniques for classifying yet unclassified drugs into ATC classes (7). To verify their method, they used classified drugs with an indication on the cardiovascular system (ATC class ‘C’). Another approach by Chen *et al.* joins chemical–chemical interaction with chemical–chemical similarity information (8) to classify drugs. Using this approach, the authors analyzed the identification of drugs among the 14 main ATC classes. Furthermore, Wang *et al.* presented NetPredATC, a drug–target network based on support vector machines for predicting the ATC class of a compound (9). They assume that drugs with similar chemical structures or target proteins share common ATC codes. Based on their assumption, they integrated the compounds chemical similarity with target information and used a support vector machine approach for the ATC code prediction. The method validation was carried out using four different drug datasets which include enzymes, ion channels (IC), G-protein coupled receptors (GPCR) and nuclear receptors (NR) as target proteins.

Recently, drug promiscuity has become an important issue in drug discovery. It was observed that drugs show a more promiscuous way of binding than it was assumed in the past (10). Due to the more complex nature of drug binding, the view of drugs as specific ligands to targets had to be reconsidered. Drug promiscuity, which entails unwanted side effects due to binding to off-targets (11), is considered as one of the main reasons for failure and withdrawal of marketed drugs. A case example represents the withdrawal of the drug combination fenfluramine/phentermine (fen–phen) because of inducing valvular heart diseases (12). Predicting targets as well as off-targets for drugs or drug candidates might help avoiding unwanted side effects as well as facilitating drug-repositioning. Several approaches have been introduced for predicting drug–target interactions. Network-based methods have been proposed to identify protein targets for drugs (13–15). Moreover, the similarity ensemble approach (16) has been proposed. The method is based on the stochastic analysis of the 2D similarity between ligands that bind to the same target and predicts ligand–target interactions adapting concepts of the basic local alignment search tool (BLAST) algorithm (17). Another method for predicting compound–target interactions is SPiDER (18). It addresses the issue of predicting targets for *de novo* designed molecules and drugs using two self-organizing maps (SOM) differing in the molecular representations for the SOM projections. The resulting two confidence scores are converted into a consensus score and contemplated in a statistical analysis to indicate the significance of the prediction.

The SuperPred web server comprises two methods, one for drug classification based on approved drugs classified by WHO (1) and one for target prediction based on compound–target interaction data. The drug classification method takes into account 2D- and fragment-similarity, and a method for 3D superposition of small molecules. The method for target prediction uses the similarity distribution among ligands for estimating the targets' individual thresholds and probabilities to avoid false positive predictions.

## MATERIALS AND METHODS

### Data set for drug classification

For drug classification, a dataset containing 2650 drugs is taken from Transformer (19). To ensure comparability between SuperPred I (20) and SuperPred II, the dataset (1035 drugs) described in SuperPred I was used for evaluation of the drug classification method.

Based on the actual drugs classified by WHO, an external dataset containing 190 novel drugs was created for validation of the drug classification method.

### Data set for the target prediction

The dataset for target prediction was created by extracting compound–target interaction data from SuperTarget, ChEMBL and BindingDB (21–23). Those databases offer a huge amount on publicly available ligand–target interaction data. To integrate the extracted data into one consistent set, several normalization steps were accomplished concerning compound and target entities and interaction data.

First, compound structures were normalized using JChem (Instant JChem 6.2.0 (January 2014), ChemAxon (http://www.chemaxon.com)). Normalization steps involved isolation of the largest fragment in the structure, removal of salts and explicit hydrogens and the standardization of stereochemical and charge information using the JChem standardization protocol. Furthermore, the structures were aromatized and formal charges were removed. For compound unification, International Chemical Identifiers (InChI) were calculated using Open Babel (http://openbabel.org/) and compounds having identical InChI were merged. Second, non-molecular target types (ChEMBL) like cell-lines, tissues and organisms and molecular target types like deoxyribonucleic acid as well as non-mammal enzymes and proteins were removed, yielding a target dataset of mammal proteins only. The remaining targets were unified using the Entrez Gene Index from NCBI (National Center for Biotechnology Information) (24) and those mapping to the same gene were merged. Third, the interaction data was filtered for certain binding types (e.g. IC50, Ki and KD), resulting in 1 900 000 interactions. Additionally, interactions described by binding affinities weaker than 10 000 nm were removed. Finally, targets having interaction data for less than five compounds were removed, resulting in a dataset consisting of ∼341 000 compounds, ∼1800 targets and ∼665 000 compound–target interactions.

For the evaluation of the target prediction, the dataset was restricted to ‘successful targets' from the Therapeutic Target Database (TTD) (25) narrowing the set to 221 targets, 95 000 compounds and 174 000 compound–target interactions.

### Drug classification pipeline

**Figure 1.**
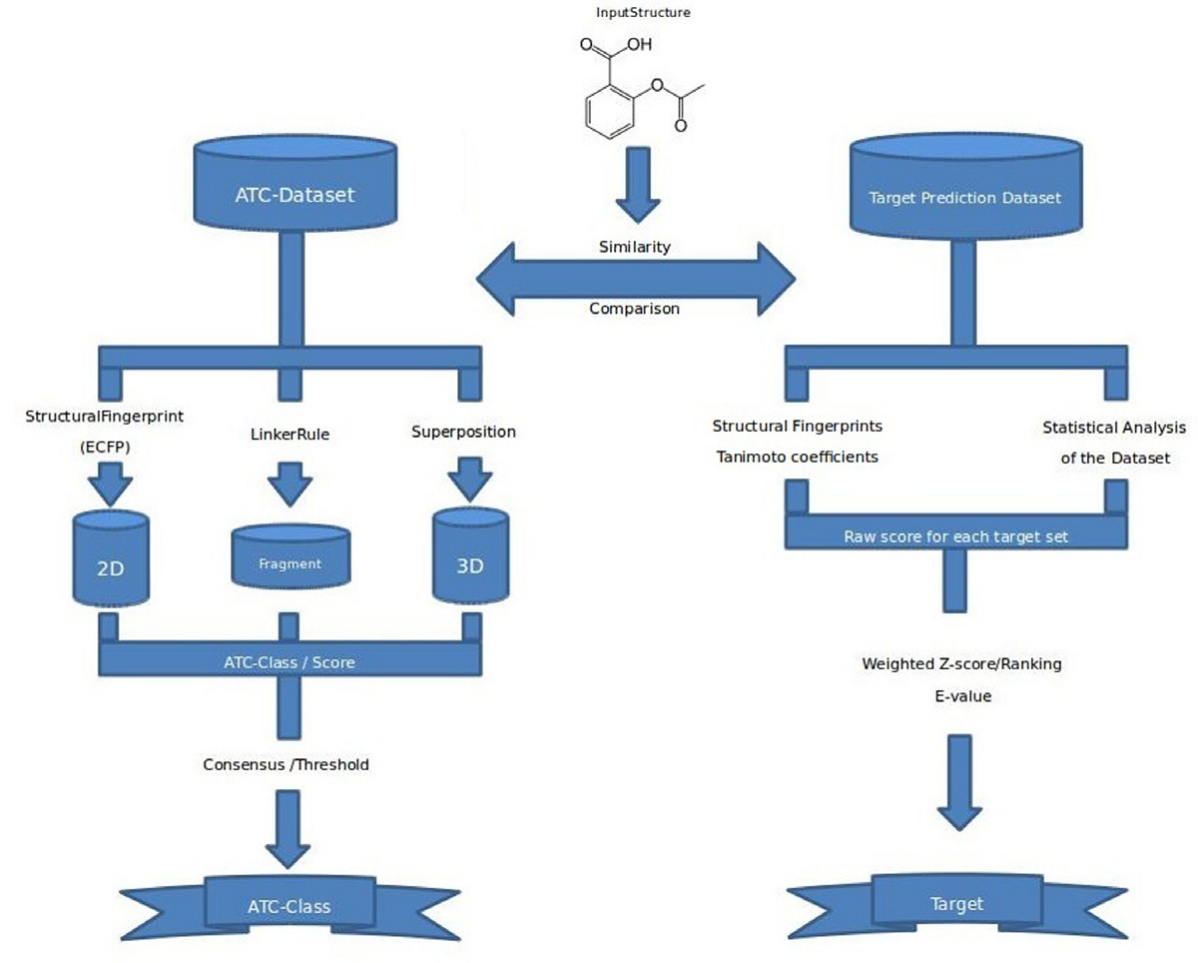
This diagram illustrates the drug classification pipeline (left) and target prediction pipeline (right). The drug classification is carried out in three steps. In the first step the input compound is compared with the ATC dataset by the following methods namely 2D, fragment and 3D similarity searching. In the second step the ATC-class and the corresponding score is calculated for each method. The last step ensembles the predicted ATC-classes according to the score and predict the final ATC-class. Similarly, the target prediction is also carried out in three main steps. In the first step, the input compound is compared based on structural similarity (2D). The second step analyzed the statistical significance of the similarity score in comparison with precalculated statistics of the dataset. The last step computes the raw score for each target and finally the target is predicted with consideration of the weighted *Z*-score and *E*-value threshold.

The drug classification pipeline is a combination of three different structure based methods, considering 2D, fragment and 3D similarity, described in detail below. This combination ensures an optimal coverage of the structural features represented by a final score. The consensus of these methods is taken into account. If at least two methods predict the same ATC class, that class is considered as final prediction. If three different ATC classes are predicted, a threshold for every method is used to decide for the most probable ATC class (Figure 1: left).

### 2D similarity searching

In order to select the optimal fingerprint for the 2D similarity comparison, several fingerprints have been compared (Table 1). The extended-connectivity fingerprints (ECFP) (26) exhibit the best performance for our dataset and hence, have been used in the prediction pipeline. The fingerprints belong to the class of radial fingerprints and are generated by a modified version of the Morgan Algorithm (27). The calculated fingerprints were subsequently compared by the Tanimoto similarity measure for bit strings (28).

**Table 1.** Comparison of fingerprints and their attained prediction rate for the evaluation dataset of 1035 compounds

Fingerprint	2D prediction rate
FP24	62.6
MDL(166)	72.3
ECFP4	74.1

### Fragment similarity searching

All 2650 drugs from the prediction dataset have been fragmented according to the linker rule (29). This method preferentially generates cyclic fragments by removing the linker atoms between ring structures. All non-redundant fragments which were produced by the fragmentation method are considered for comparison. While comparing the fragments of two small molecules (A and B) having *n* and *m* fragments, a similarity matrix with *n* × *m* fields is constructed. Each field contains the Tanimoto coefficient of the particular fragment comparison. The matrix is used to calculate $\left( {\frac{n}{m}} \right)$ possible fragment combinations. For each combination, a final Tanimoto score is calculated by summing up its Tanimoto coefficients from the matrix. The final similarity score is further divided by the smaller number of fragments belonging to one of the molecules.

### 3D similarity searching

The superimposition of one molecule to a reference molecule structure is done by mapping atoms with optimal distances. In order to reduce time complexity, only 100 low-energy conformations are generated for the two molecules to be compared and pairwise comparisons of all possible conformations are performed. Hence, given a molecule pair, a maximum of 10 000 comparisons take place (30). The first step of the algorithm normalizes the set of atoms into a new coordinate system. Based on these coordinates, the centers of mass for both conformers are calculated and superimposed. Then, the principal axes of inertia are estimated and aligned. Thereby, the possible rotations are strongly reduced and only four orientations have to be considered. For every orientation, a mapping of atom pairs is performed whereupon atoms are fitted to each other with the smallest possible distance. A maximal distance threshold is applied for atom pair assignment, therefore not every atom is assigned. The rotation matrix with the highest amount of mapped pairs was used for further calculations. The normalized variant with the minimal distance is chosen if more than one rotation with the same amount of mapped atom pairs exists. For this mapping a root-mean-square-deviation (rmsd) was calculated. To find the best superposition of two molecules, the number of superposed atoms and the corresponding rmsd value are taken into account by the following formula:

\begin{equation*}
3{\rm D}-{\rm score} = \frac{{N_{\rm S}}}{{\max (N_{\rm A} \times N_{\rm B})}} \exp ( - {\rm rmsd})
\end{equation*}

where *N*_S_ is the number of superposed atoms, *N*_A_ the number of atoms of molecule A and *N*_B_ the number of atoms of molecule B.

### Target prediction method

The method for the drug–target prediction takes into consideration the 2D similarity between the query compound and the ligands associated to their respective targets (target sets). For each target set, the summation of all Tanimoto coefficients above a threshold of 0.45 is considered as raw score. To achieve comparability between raw scores of small and large target sets, the raw scores are normalized by dividing them by the number of ligands of the corresponding target. To further evaluate the specificity of a prediction, *Z*-scores and *E*-values are computed. The *Z*-score is calculated by the formula:

\begin{equation*}
Z_{\rm A} = \frac{{\left( {\frac{{({\rm raw}\,{\rm score}_{\rm A} )}}{{N_{\rm A} }} - \mu } \right)\exp (0.335\ln (N_{\rm A} ))}}{\sigma }
\end{equation*}

where A is a target set and *N*_A_ represents the number of ligands of target set A. Similar to BLAST (17) *μ* and *σ* describe the random background noise of the database.

The *E*-value describes the number of predicted targets one can expect to see by chance, thereby it depends on the size of the dataset. The *E*-value decreases exponentially as the *Z*-score of the prediction increases. The lower the *E*-value, the more significant is the prediction (17). (For further details and formulas please see the FAQ section on our SuperPred website).

For diverse target sets, *Z*-scores tend to behave like high random scores. Therefore, a weighting factor *λ*_A_ is introduced which indicates the average similarity between the ligands within each target set:

\begin{equation*}
\lambda _{\rm A} = \exp \left( {0.335\ln \left( {\frac{{{\rm raw}\,{\rm score}_{{\rm AA}} }}{{N_{{\rm AA}} }}} \right)} \right)
\end{equation*}

The weighting factor ranges between almost one for very uniform target sets to more than ten for very diverse target sets. The target prediction results are ranked according to the weighted *Z*-scores (Figure 1: right).

### Input and output options

There are four input options available for drug classification and target prediction. First, via the ChemDoodle tool (http://www.chemdoodle.com/), an upload function for MOL files is provided. Second, it is possible to draw a structure using the ChemDoodle editor. Third, a PubChem (31) name search option is provided and fourth, a molecule can be searched by its Simplified Molecular Input Line Entry Specification (SMILES) (http://daylight.com/smiles/).

The output for the ‘Drug Classification’ and the ‘Target-Prediction’ displays the input compound's properties and its molecular structure. In case of the ‘Drug Classification’ result site the prediction accuracy, the ATC-class and information about similar drugs, that have ATC-codes assigned, is given. Furthermore, Lipinski-rule of five properties (32) for the uploaded compound are also shown. In addition, the statistics for physico-chemical properties for the predicted ATC class are presented. Moreover, a button is provided to start the target prediction for the input compound likewise the ‘Target-Prediction’ result site offers a button for starting the drug classification. Furthermore, it displays known and predicted targets for the input compound and provides detailed information about the targets. Links to other databases as well as available PDB structures are given.

## RESULTS AND DISCUSSION

### Drug classification

**Figure 2.**
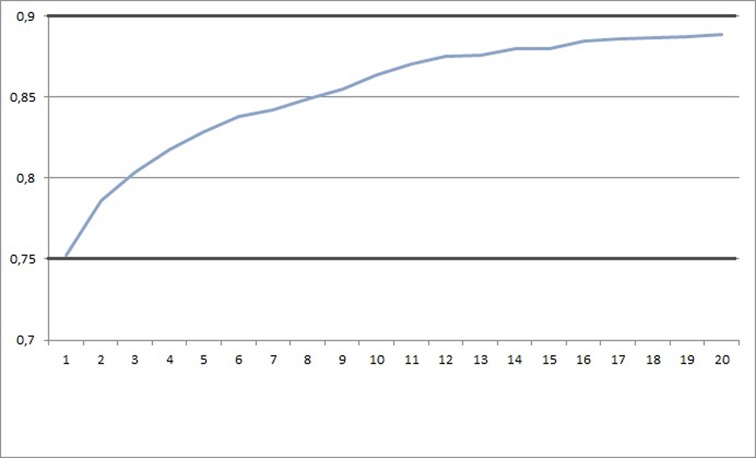
Cumulative recall graph for ATC-prediction relative to the rank of retrieved compounds.

The implementation of the new prediction pipeline (Figure 1: left) consisting of 2D, fragment and 3D similarity searching methods results in a higher prediction rate for ATC classes compared to SuperPred I based on the evaluation dataset from SuperPred I. In SuperPred II, the prediction accuracy has increased to 75.1% for the validation set (Table 2). SuperPred I, taking only into account the 2D similarity of compounds, showed a prediction accuracy of 67.6%. The prediction rates’ distribution values of the correctly predicted ATC codes are shown in Table 3. For a consensus score range between 0.8 and 0.9, a prediction rate of 88.9% is achieved. Furthermore, a cumulative recall graph is shown in Figure 2 representing the fraction of correct ATC class predictions in dependency of the quantity of retrieved molecules. By taking into account the three most similar structures, a prediction rate of 80.3% is reached whereas a recall of maximal 88% is reached by taking at least 16 similar compounds into consideration. For further validation of the prediction pipeline, we utilized the 190 new drugs contained in the evaluation dataset. The prediction accuracy for this dataset is 72.1% .

**Table 2.** Prediction accuracy overview of the individual drug classification methods (2D, fragment, 3D similarity methods) as well as for the combined pipeline (consensus) for the evaluation dataset

Method	Prediction accuracy (%)
2D	74.1
Fragment	69.4
3D	67.7
Consensus	75.1

**Table 3.** Prediction rate distribution for correctly predicted ATC codes

Range of consensus score	Number of hits/misses	Prediction rate (%)
0.0–0.1	0/0	0
0.1–0.2	2/4	33.3
0.2–0.3	6/40	13.0
0.3–0.4	14/22	38.9
0.4–0.5	20/43	31.8
0.5–0.6	50/32	61.0
0.6–0.7	88/24	78.6
0.7–0.8	164/36	82.0
0.8–0.9	233/29	88.9
0.9–1.0	199/27	88.1

The distribution is based on the evaluation dataset, which contains 1035 drugs. This table shows the number of right (hits) and wrong (misses) predictions for a specific consensus score range.

### Comparison to other drug classification methods

In comparison to other drug classification methods, SuperPred II yields the best prediction rate (Table 4). Chen *et al.* have analyzed their prediction performance of the first level of ATC codes (13). They have combined chemical–chemical interaction with chemical–chemical similarity information. Based on this, their method reaches a prediction rate of 73.25% for prediction of the 14 main ATC classes. To make our method comparable, we modified our prediction to the first level ATC class. This resulted in a prediction rate of 80.9% for identifying the right ATC classes among the 14 main classes.

**Table 4.** Comparison of the SuperPred update with other ATC prediction methods

	Total accuracy [%]	Comment
SuperPred (2008)	67.6	Overall prediction
NetPredATC (2013)	74.0	GPCR
Chen et. Al (2012)	73.25	Main ATC classes
SuperPred (2014)	75.1	Overall prediction

The comment column indicates how the prediction accuracy was achieved.

Furthermore, we compared our method with NetPredATC from Wang *et al.* The accuracy of NetPredATC lies between 74 and 76.5% according to the previously mentioned subsets belonging to single target classes like GPCR, NR, IC and enzymes.

### Target prediction

The target prediction method, results in a prediction accuracy of 91.2% without the use of the weight function (*λ*). Considering the weight function, the prediction rate increases to 92.8%. Additionally, the *E*-value is used as a threshold: an *E*-value above 1 is an indication of random prediction. Considering this threshold, it was observed that the prediction rate further increases to 94.1%. However, about 9400 compounds were not considered for prediction because they were above the *E*-value threshold. It was also observed that the target groups which have more diverse compounds show lower prediction rates. This could be caused by multiple binding sites on the target or by targets with different domains that have different properties and bind different types of ligands, resulting in subsets of related compounds inside its target set.

## CONCLUSION

In comparison to the ATC prediction method described by Chen *et al.*, the SuperPred II method is able to produce a higher prediction accuracy of ∼8%. Wang *et al.* perform their prediction on four relatively small benchmark datasets whereas SuperPred II considers a wide range of target classes and produces a comparable prediction accuracy. For further improvement of the drug classification method an integration of drug-protein networks could increase the prediction accuracy as drug pairs having the same ATC code may bind to the same targets (33).

To improve the target prediction method, target groups with diverse compounds due to multiple binding sites or different domains will be considered as independent target sets as agonists and antagonists bind to different binding sites or domains and cause different pharmacological effects.
